# Restoring Cognitive functions: Unveiling the Procognitive and Neurotrophic Impact of Chronic Administration of a Selective A5‐GABAA Receptor Positive Allosteric Modulator in Mouse Models of Amyloid Load and Tau Phosphorylation

**DOI:** 10.1002/alz.088385

**Published:** 2025-01-09

**Authors:** Thomas D Prevot, Ashley D Bernardo, Kayla D Wong, Celeste Pina‐Leblanc, Michael D Marcotte, Dishary Sharmin, Prithu Mondal, James M Cook, Etienne Sibille

**Affiliations:** ^1^ Centre for Addiction and Mental Health, Toronto, ON Canada; ^2^ University of Wisconsin ‐ Milwaukee, Milwaukee, WI USA

## Abstract

**Background:**

Dysregulated GABA/somatostatin (SST) signaling has been implicated in psychiatric and neurodegenerative disorders. The inhibition of excitatory neurons by SST+ interneurons, particularly through α5‐containing GABAA receptors (α5‐GABAAR), plays a crucial role in mitigating cognitive functions. Previous research demonstrated that an α5‐positive allosteric modulator (α5‐PAM) mitigates working memory deficits and reverses neuronal atrophy in aged mice. Subsequently, we explored the behavioral and neurotrophic effects of this α5‐PAM in animal models reflecting β‐amyloid load and tau phosphorylation, hallmarks of Alzheimer’s disease progression.

**Method:**

Two studies were conducted with approximately 12 mice per group, including 50% females: 1) 5xFAD transgenic mice exhibiting progressive amyloid‐related cognitive decline; 2) PS19 transgenic mice displaying progressive tau‐tangle‐cognitive decline. Chronic administration of GL‐II‐73 (30mg/kg, p.o, for 4 weeks) was employed to assess its efficacy in rescuing cognitive deficits across three domains. Working memory, spatial memory, and cognitive flexibility were evaluated through an alternation task, water maze, and set‐shifting assay, respectively. Golgi‐Cox staining of brains (n = 4brain/group; 8cell/brain) facilitated the quantification of dendritic length and spine density in the prefrontal cortex and hippocampus using NeuroLucida. In the 5xFAD cohort, the impact of treatment on spine maturation steps was also examined.

**Result:**

Chronic treatment in both models reversed cognitive deficits across domains (ps<0.01), demonstrating a robust effect on working memory. Furthermore, it significantly mitigated amyloid‐induced and tau‐related dendritic shrinkage and reduction of spine density at apical and basal dendrites (p<0.001 in PFC and CA1). Notably, this effect extended to all maturation steps in the 5xFAD model.

**Conclusion:**

The findings collectively indicate that selectively targeting α5‐GABAA receptors overcomes amyloid‐ or tau‐related cognitive deficits and neuronal morphology detriments. This marks the first intervention addressing the GABAergic system with both symptomatic and disease‐modifying therapeutic potential across various disorders. This breakthrough suggests a promising avenue for clinical development, offering hope to patients grappling with cognitive deficits across a spectrum of brain disorders.

